# Comment on: Filippini, T.; Tesauro, M.; Fiore, M.; Malagoli, C.; Consonni, M.; Violi, F.; Iacuzio, L.; Arcolin, E.; Oliveri Conti, G.; Cristaldi, A.; Zuccarello, P.; Zucchi, E.; Mazzini, L.; Pisano, F.; Gagliardi, I.; Patti, F.; Mandrioli, J.; Ferrante, M.; Vinceti, M. Environmental and Occupational Risk Factors of Amyotrophic Lateral Sclerosis: A Population-Based Case-Control Study. *Int. J. Environ. Res. Public Health* 2020, *17*, 2882

**DOI:** 10.3390/ijerph17186490

**Published:** 2020-09-07

**Authors:** Aisha S. Dickerson

**Affiliations:** Department of Epidemiology, Johns Hopkins Bloomberg School of Public Health, Baltimore, MD 21205, USA; adicke10@jhu.edu

A recent paper was published by Filippini et al. in the *International Journal of Environmental Research and Public Health* (22 April 2020) reporting the results of a population-based case-control study of environmental and occupational exposures and risk of amyotrophic lateral sclerosis (ALS) in four provinces in Italy [1]. Given my similar research in this area using population-based Danish registry data to ascertain all ALS cases diagnosed in Denmark from 1982 to 2013 and lifetime occupation history, I took special interest in this manuscript and would therefore like to make some contributions to the portion of the authors’ discussion on risk associated with occupational sectors and specific occupational exposures.

The results for the Filippini et al. paper demonstrate slightly increased, but not close to statistically significant, risk of ALS in men working in the agricultural sector (adjusted odds ratio (aOR) = 2.09, 95% confidence interval (CI) 0.79–7.54) [1]. Although these results are consistent with previous studies, including those of our study which reported higher odds of ALS for men working in agriculture and farming (aOR = 1.20, 95% CI 0.99–1.44) [2], it is important to note that the small sample size of the Filippini et al. study, acquired from 4 provinces in Italy, may lend itself to significantly reduced power with only nine ALS cases and eight controls reporting work in this sector [1].

The authors also found significantly increased risk of ALS in men with any occupational exposure to lead (aOR = 3.66, 95% CI 1.63–8.20) [1]. However, these exposures were not lagged to assess potential differences in windows of exposures. This is particularly interesting considering that in our analysis of occupational lead exposure in Denmark, we observed stronger associations in those with higher cumulative lead exposure, high probability (≥50%) of exposure, and exposures limited to occupations held 5 and 10 years before the ALS diagnosis date [3]. Considering that lead can accumulate over several years, store in bone, and later mobilize to neural tissues [4], in addition to the likely lengthy latency period for ALS symptomology and subsequent diagnosis, I believe it is important to examine earlier windows of exposure, especially in the case of lead and ALS.

Lastly, I was pleased to see that the authors’ results for associations with solvents were consistent with those from our Danish population, which found no statistically significant association with overall exposure to solvents in males [5]. Notably, the Filippini et al., manuscript did observe positive associations of ALS risk with paint thinners (aOR = 2.27, 95% CI 1.14–4.54) and removers (aOR = 2.01, 95% CI 0.90–4.48) [1]. These results are also in line with our results showing increased ALS risk in men exposed to methylene chloride (aOR = 1.23, 95% CI 1.07–1.42, *P* = 0.003), most commonly present in paint strippers, at least 5 years prior to ALS diagnosis [5]. Furthermore, when we examined solvent mixtures in those with any solvent exposures, weighted quantile sum (WQS) analysis revealed that the greatest weight of ALS risk was contributed to methylene chloride exposure [5], which highlights the importance of parsing out specific environmental chemicals and using analyses to evaluate attributable impact and potential interactions of concurrent exposures.

As the authors mention, their results are restricted to cases with sporadic ALS, which our analysis in Denmark was unable to do, but the recruitment and exposure assessment methods of this particular study introduce some important biases that may limit the comparability of results from these two European populations. Nevertheless, I am intrigued by the authors’ ability to assess co-exposures in this Italian population and look forward to reading more on the ongoing research from this group.
